# Three-Dimensional 3D Culture Models in Gynecological and Breast Cancer Research

**DOI:** 10.3389/fonc.2022.826113

**Published:** 2022-05-26

**Authors:** Yarely M. Salinas-Vera, Jesús Valdés, Yussel Pérez-Navarro, Gilberto Mandujano-Lazaro, Laurence A. Marchat, Rosalio Ramos-Payán, Stephanie I. Nuñez-Olvera, Carlos Pérez-Plascencia, César López-Camarillo

**Affiliations:** ^1^ Departamento de Bioquímica, Centro de Investigación de Estudios Avanzados (CINVESTAV-IPN), Ciudad de Mexico, Mexico; ^2^ Posgrado en Ciencias Genómicas, Universidad Autónoma de la Ciudad de Mexico, Ciudad de Mexico, Mexico; ^3^ Programa en Biomedicina Molecular y Red de Biotecnología, Instituto Politécnico Nacional, Ciudad de Mexico, Mexico; ^4^ Facultad de Ciencias Químico Biológicas, Universidad Autónoma de Sinaloa, Culiacán Sinaloa, Mexico; ^5^ Departamento de Biología Celular y Fisiología, Instituto de Investigaciones Biomédicas, Universidad Nacional Autónoma de México, Ciudad de México, Mexico; ^6^ Laboratorio de Genómica, Instituto Nacional de Cancerología, Ciudad de México, Mexico

**Keywords:** 3D cultures, breast cancer, gynecological cancers, microRNAs, therapy response

## Abstract

Traditional two-dimensional (2D) monolayer cell cultures have long been the gold standard for cancer biology research. However, their ability to accurately reflect the molecular mechanisms of tumors occurring *in vivo* is limited. Recent development of three-dimensional (3D) cell culture models facilitate the possibility to better recapitulate several of the biological and molecular characteristics of tumors *in vivo*, such as cancer cells heterogeneity, cell-extracellular matrix interactions, development of a hypoxic microenvironment, signaling pathway activities depending on contacts with extracellular matrix, differential growth kinetics, more accurate drugs response, and specific gene expression and epigenetic patterns. In this review, we discuss the utilization of different types of 3D culture models including spheroids, organotypic models and patient-derived organoids in gynecologic cancers research, as well as its potential applications in oncological research mainly for screening drugs with major physiological and clinical relevance. Moreover, microRNAs regulation of cancer hallmarks in 3D cell cultures from different types of cancers is discussed.

## Introduction

**Figure 1:**
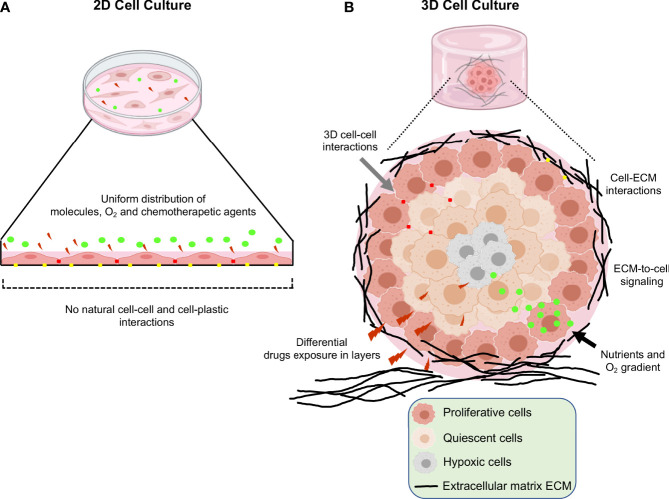
Schematic representation of the main differences between 2D and 3D cell cultures. **(A)** Traditional 2D cell culture in which flattened cells grown in a monolayer at the bottom of plastic plates. Reduced cell-cell interactions, unlimited exposure to nutrients, oxygen and drugs are limitations of this type of cultures. **(B)** 3D cell culture systems; in which increased cell-cell and cell-extracellular matrix interactions, limited access to nutrients, oxygen, and heterogeneity in the drugs interactions leads to better recapitulation of the tumor microenvironment occurring *in vivo*.

Three-dimensional (3D) cell cultures are a breakthrough for gynecological and breast cancer research as they mimic the 3D architecture of primary tumors. For a long time, oncology research was based on 2D monolayer cultures, where cells grown on a flat solid surface. However, this culture model has limitations, such as the absence of cell-cell and cell- extracellular matrix (ECM) interactions, and tumor microenvironment, as well as unlimited access to nutrients, oxygen, and metabolites (1, 2). Additionally, cells cultured in 2D modify their morphology and cause cytoskeletal rearrangements, acquiring artificial polarity, which in turn leads to aberrant gene and protein expression (**Figure 1A**) (3, 4). On the other hand, 3D cultures promote cell-cell and cell-ECM interactions (5). This culture model better recapitulates the characteristics of tumor cells *in vivo*, such as cell heterogeneity, hypoxia, growth kinetics, signaling pathway activity and gene expression patterns (6, 7). Moreover, in 3D cultures the morphology and polarity of tumor cells are maintained, and a concentration gradient of O_2_, nutrients and metabolic waste is generated, making them an ideal model to study tumor cells behavior (**Figure 1B**) (8, 9). Several reports showed the advantages of using 3D culture systems for gynecological cancer studies, as they allow the evaluation of the effect of the extracellular matrix on the tumor, reducing the existing breach between 3D culture models and *in vivo* models (**Table 1**).

**Table 1:** Advantages and disadvantages of using 3D versus 2D culture.

Characteristic	2D	3D			Reference
		Spheroids	Organotypic	Organoid	
Support	Plastic, polycarbonate	Low-adherence plastic plates	Extracellular matrix *in vitro*	Extracellular matrix *in vitro*	(10, 11)
Duration of cultivation	long-term culture	Short-term culture	Short-term culture	Robust and stable in long-term culture	(12)
Interaction and communication	N/A	Cell-cell interactions	Cell-cell and cell-matrix 3D interactions	Cell-cell, cell-stroma and cell-matrix 3D interactions	(13)
Cell forms	Flat and extensible	Natural cellular structure preserved	Natural cellular structure preserved	Natural cellular structure preserved	(14)
Cell junctions	Less common	More common (cell-cell communication)	More common (cell-cell communication)	More common (cell-cell communication)	(2)
Maintain	Easy to maintain and passage	Easy to maintain	Easy to maintain	Difficult to maintain and expensive	(12)
Drug response	Cells more sensitive to treatment	Cells more sensitive to treatment	Cells less sensitive to treatment	Cells less sensitive to treatment	(6, 8)
Reproducibility	High reproducibility	High reproducibility	High reproducibility	Lack of reproducibility due to patient heterogeneity	(9)

The development of 3D cultures and its more generalized utilization have permitted the evaluation of changes in gene expression mechanisms relative to 2D conditions, mainly in mRNA transcriptomes. However, scarce data on postranscriptional control of gene expression represented by microRNAs (miRNAs) have been studied in 3D cancer cell cultures. MiRNAs are small non-coding RNAs of about 21-25 nucleotides in length that function as negative regulators of gene expression at the post-transcriptional level (15). The miRNAs contain a seed region corresponding to 2-7 nucleotides, which binds by bases complementarity to conserved sites in the 3’ untranslated regions (UTR) of target mRNAs, resulting in mRNA degradation or translation repression (16). Increasing evidence shows that miRNAs regulate diverse processes involved in cancer progression, such as, cell proliferation, apoptosis, invasion, metastasis, and drug resistance (17, 18). Due to their high stability, miRNAs are also being tested in clinical trials as therapeutic agents for treatment of oncological patients (19). The objective of this review is to address the 3D modeling systems in cancer research, as well as potential applications in gynecological and breast cancers, because they are main oncological diseases affecting the female population. Finally, the differential regulation of miRNAs in 3D cultured breast and gynecological cancer cells is addressed, in order to understand its biological functions and if they could be potential therapeutic targets.

## Types of 3D Culture Systems in Cancer

Nowadays, 3D culture systems are divided into three categories: spheroids, organotypic cultures and organoid models. Spheroids are commonly referred to cultures in which cancer cell lines grown in low-adherence plastic plates or over inert substrates like agarose with continuous agitation, in which no ECM is utilized as substrates. In contrast, 3D organotypic cultures of cancer cell lines are *in vitro* systems in which the cells are cultured on commercial matrigel containing extracellular matrix proteins which provides a semi-solid support simulating some features of the *in vivo* tumor microenvironment such as cell-cell and cell-ECM interactions which activate cell signaling. On the other hand, the organoids which are generally *ex vivo* systems mainly patient-derived explants (PDE), are cultured on matrigel that simulate the extracellular matrix and facilitate drug testing in intact human tumors. In the next sections, we will discuss the different types of 3D culture systems.

### Spheroids Models

**Figure 2:**
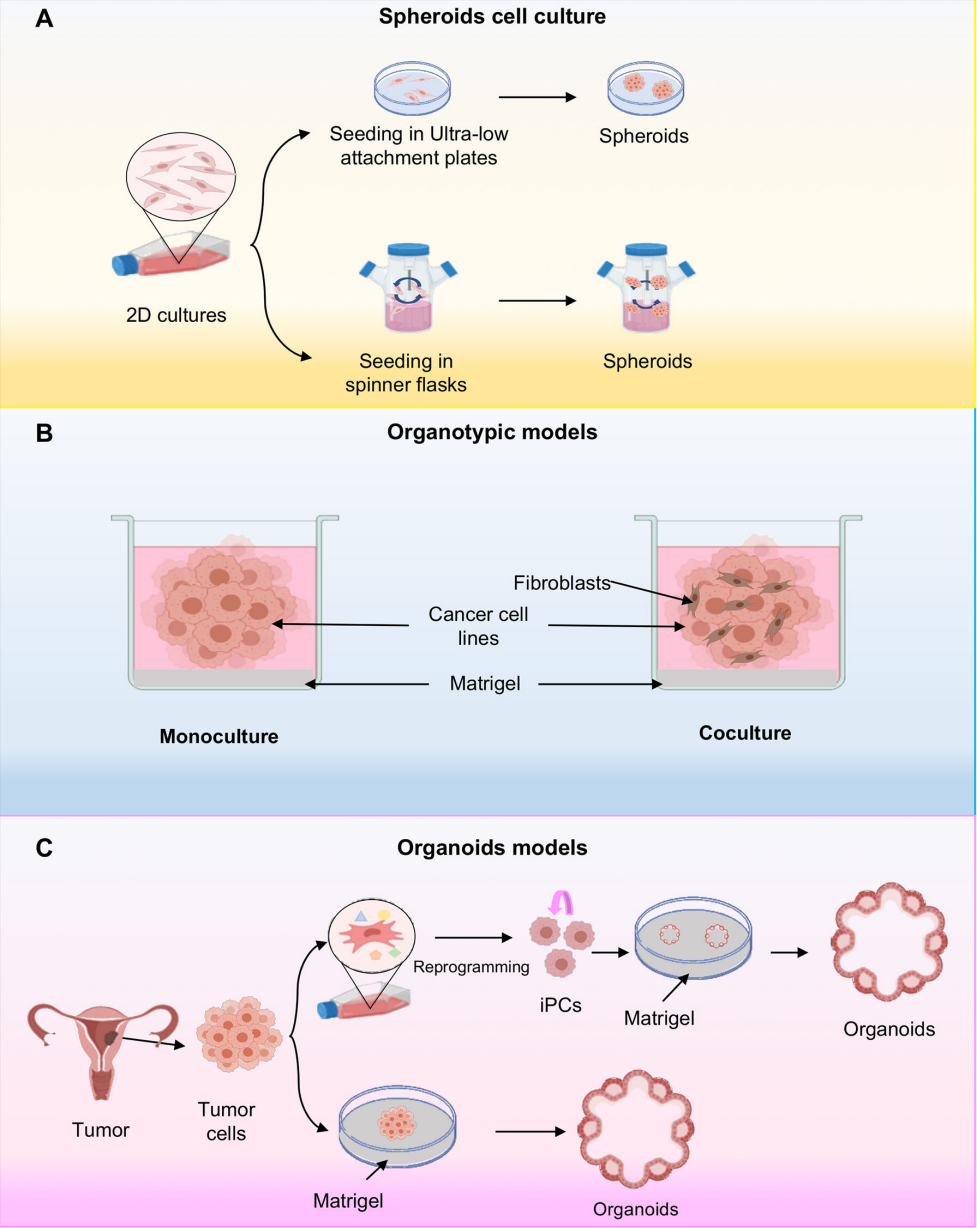
Three-dimensional cell cultures. **(A)** Scheme representing the cellular spheroids grown in ultra-low attachment plates. In this system, cancer cells are deposited on an ultralow fixation plate that prevents sticking and allowing the grown of cells in suspension; or alternatively they are placed in spinning flasks and subjected to gravitational forces also inducing the spheroids formation. **(B)** Schematic representing organotypic models, where organotypic models have been generated in monoculture or in combination with fibroblasts cocultures. **(C)** Representative schematic of organoid establishment from pluripotent stem cells (iPSCs) and cancer cells. The iPSCs first undergo reprogramming, followed by directed differentiation, and are then seeded into an extracellular matrix in a specific culture medium to initiate organoid culture. The tumor tissue organoids were processed to remove excess fat and necrotic cells and cut into small pieces. They are then seeded on Matrigel.

Spheroids are cell aggregates that can be grown in suspension, for example on low-adhesion plastic plates or over inert substrates such as agarose with continuous agitation without the presence of matrigel (11). The suspension culture method was developed in 1970 by Sutherland and coworkers (20). They used an *in vitro* 3D model system to recreate the complexities of the multicellular tumor to study the response of tumor cells to radiotherapy. In this method, ultra-low attachment plates are used or standard plastic plates coated with inert substrates, for example, agar or poly-2-hydroxyethyl methacrylate (poly-HEMA), which prevents cells from adhering to the surface of the wells, forcing cells to aggregate and form spheroids (21). On the other hand, the system from spinner flasks consists of cells suspension and a shaker element that maintains continuous movement. The liquid flow not only prevents cell adhesion, but also ensures uniform distribution of nutrients and oxygen in cells. This method produces high yields of spheroids (22, 23) (**Figure 2A**).

### Organotypic Cultures of Cancer Cell Lines

The 3D culture systems or organotypic models are generated by *in vitro* culturing cancer cell lines in a semisolid extracellular matrix under defined culture medium conditions (**Figure 2B**) (24, 25). They are an attractive model as they recapitulate the characteristics of tumor cells *in vivo* with respect to growth kinetics, cellular heterogeneity, signaling pathway activity. Additionally, it has been shown that gene expression in 3D cultures is much closer to clinical expression profiles than those observed in traditional 2D monolayer culture (6). Interestingly, organotypic cultures show diverse morphologies depending on the inherent nature of the cell and culture conditions, for example, in breast cancer three different morphologies have been observed depending on the molecular subtype of the cell line, such as mass, grape bunch and stellate (26). Moreover, to further understand cancer biology, they have developed 3D co-culture models in order to effectively model the influences of the tumor microenvironment on drug efficacy. Thus, organotypic models possess features more appropriate for high-throughput screening assays compared to 2D conditions (9) (**Figure 2B**).

### Organoid Models

Organoids are 3D systems that have been established for cancer research as they recapitulate the genotype, phenotype and cellular behavior of parental tissues (27). These innovative cultures models can be developed from both induced pluripotent stem cells (iPSCs), and tumor tissues (28, 29). Organoids established from induced pluripotent stem cells (iPSCs) begins with the isolation and culture of malignant cells from a primary or metastatic tumor sample (30, 31) (**Figure 2C**). Subsequently, reprogramming is carried out through gene transfer of SOX2, KLF4, c-MYC, and OCT4 transcription factors by means of retroviruses or lentiviruses (32). These cells then differentiate into the cell type of origin of the initial tumor. Differentiated iPSC-derived cells can be used to derive organoids. However, iPSC-derived organoids have major disadvantages because their efficacy depends on the type of cancer and the presence or absence of oncogenic mutations potentially selecting for the growth of tumor subclones and the loss of genetic heterogeneity of the tumor from which they are derived (33). In general, it is more practical to grow tumor organoids directly from tumor tissue. On the other hand, cancer tissue-derived organoids are established from the collection of tumor tissue after biopsy and placed on a matrigel-coated surface where it is embedded within the matrigel (34) (**Figure 2C**). The main advantage of this system is the preservation of the original tumor tissue architecture, including cellular and non-cellular components of the tumor microenvironment and cell-cell interactions However, the main disadvantage is the lack of reproducibility due to tumor heterogeneity (9, 35).

## 3D Cultures Systems in Gynecological Cancers

Traditional 2D cell cultures and animal models represent the experimental mainstay for gynecologic cancer research. However, their ability to reflect mechanisms occurring *in vivo* is limited. This is because the cellular models lack the tumor microenvironment and associated cellular interactions, which limits their application to clinical practice and research. Therefore, the development of technologies such as 3D culture will provide a novel alternative for gynecologic cancer research, as it allows to replicate several critical features of tissues including tumor morphology, differentiation, polarity, proliferation rate, gene expression, cell heterogeneity, and nutrient and oxygen gradients (2).

## 3D Cultures Systems in Cervical Cancer

**Figure 3:**
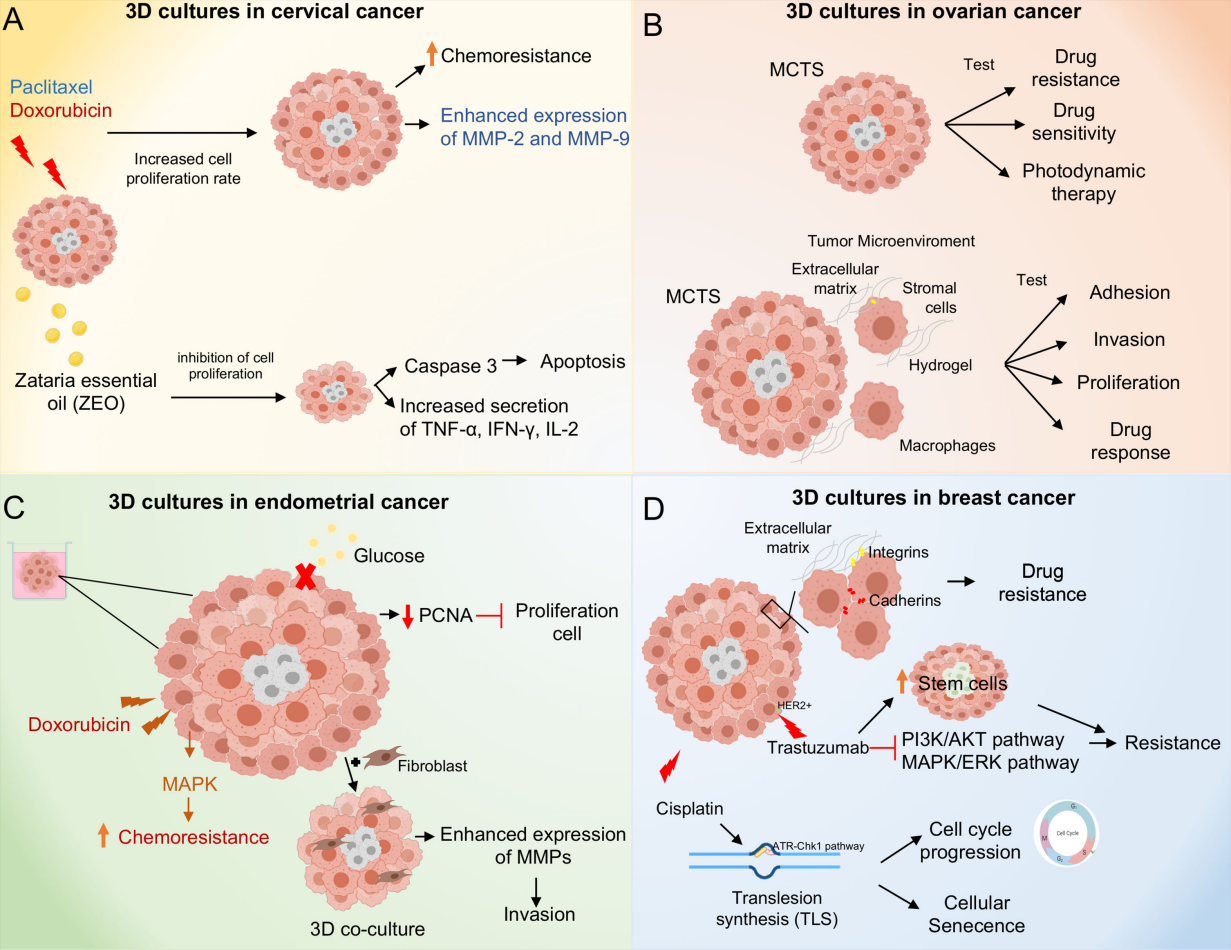
Molecular mechanisms activated in 3D culture systems. **(A)** 3D cultures of cervical cancer cells result in paclitaxel and doxorubicin chemoresistance through increased proliferation rate and overexpression of MMP-2 and 9. Zataria essential oil treatment inhibits cell proliferation in 3D cultures and induces apoptosis through activation of caspase 3. **(B)** 3D culture systems in ovarian cancer. The Multicellular Tumor Spheroids (MCTS) allow testing of drug and photodynamic therapies. The co-culture of MCTS with stromal cells and macrophages in combination with hydrogels as scaffold, allow to mimic the tumor microenvironment providing a model to test adhesion, invasion, proliferation processes as well as drug response. **(C)** 3D cultures systems in endometrial cancer. Doxorubicin treatment induces chemoresistance through activation of the MAPK pathway. Moreover, 3D cultures of endometrial cancer co-cultured with fibroblasts promote invasion through overexpression of MMPs. **(D)** 3D cultures systems in breast cancer. Spheroids of breast cancer cells exhibit cell-cell and extracellular cell-matrix interactions promoting drug resistance. Cisplatin treatment promotes cell cycle progression and cellular senescence through up-regulation of trans-lesion DNA polymerase (TLS) expression and activation of the ATR-Chk1 pathway. Trastuzumab treatment induces resistance in 3D cultures through inhibition of PI3K/AKT and ERK/MAPK pathways, in addition to an increase in stem cells subpopulations.

Cervical cancer is the fourth most common cancer among women globally and therapy resistance is still a major problem to treat the disease (36). Hence, it is necessary to develop novel drugs and therapeutic approaches, as almost all drugs used today suffer from serious side effects due to drug resistance and lack of selectivity towards tumors (37). Recently, there has been an increasing interest in the development of 3D *in vitro* tumor models based on human cancer cells to accurately reproduce the characteristics of human cancer tissues (38). For instance, Zhao and coworkers demonstrated increased paclitaxel chemoresistance and proliferation rate in 3D cultures compared to traditional monolayer (2D) cultures of HeLa cells. In addition, HeLa cells increased the expression of matrix metalloproteinase (MMP) protein in 3D cultures (39). Similarly, Baek N and coworkers demonstrated increased resistance to doxorubicin in 3D cultures of HeLa cells compared to 2D cultures, resulting in higher IC50 values 11.2 and 9.6 μM of doxorubicin in 3D cultures at day 3 and 5, respectively, compared to monolayer cultures 1.0 μM of doxorubicin. The observed differences to doxorubicin sensitivity with respect to 2D and 3D cultures is due to monolayer cultured cells being well oxygenated, resulting in the rapid accumulation of reactive oxygen species (ROS) when exposed to DXR. In contrast, cells in the spheroid core are under hypoxic conditions, which makes them much more resistant (40). It has been shown that some plant constituents have anticancer activities, for example, Zataria essential oil (ZEO) is one of the useful essential oils that possesses extensive biological activities. The major components of ZEO have been shown to decrease the viability of breast cancer cells (41). Azadi M and coworkers demonstrated that ZEO treatment promotes inhibition of cell proliferation and promotes apoptosis in the TC1 cervical cancer cell line TC1 in both monolayer (2D) and multicellular spheroids (3D). In addition, ZEO was effective in tilting the cytokine balance in favor of T helper 1 through increased secretion of TNF-α, IFN-γ, IL-2 and decreased IL-4 (**Figure 3**) (42). It has been shown that bidirectional crosstalk between tumor and stroma plays an important role in the response to therapy. De Gregorio V and coworkers developed an organotypic cervical tumor model where they established this crosstalk, they developed two models 1) composed of primary human cervical fibroblasts (HCFs) embedded in the ECM, to produce normal cervical stroma (NCIS) and 2) composed of cervical cancer-associated fibroblasts (CCAFs), generating cervical cancer stroma (CCIS). They demonstrated increased gene expression of early viral E6 and E7 genes in SiHa cells when cultured in CCSI. Therefore, organotypic models of cancer can help to better understand cancer progression and establish novel anti-cancer therapeutic targets directed to tumor stroma and cancer cells (43).

### MicroRNAs Modulation in 3D Cervical Cancer Cultures

As major regulators of gene expression, its expected that miRNAs expression could be modulated in the 3D cultures, as several studies reported (44). The miR-143/145 cluster has been found downregulated in cervical cancer and overexpression of miR-143 or miR-145 inhibits cell viability, proliferation, migration, and invasion, in monolayers and 3D cultures of HeLa cervical cancer cell line. Furthermore, transfection of miRNA-145 increased pMLC levels by targeting the MYPT1 subunit of myosin regulatory phosphatase (45). Moreover, it has been shown that extracellular vesicles (EVs) secreted by 3D cultured tumor cells differ in terms of secretion dynamics and essential signaling molecular contents (RNA and DNA) compared to EVs derived from monolayer 2D cultures. These data suggested that EV small RNAs derived from 3D cultures may reflect EVs RNAs derived from *in vivo* tissues (46). Indeed, this is because cells in monolayer completely differ from the *in vivo* state where cells grow in 3D, in terms of cell morphology, cell-to-cell interactions, growth behavior and interactions with the extracellular matrix (1, 47). Thippabhotla and coworkers demonstrated that miRNAs expression profile of extracellular vesicles derived from 3D culture of HeLa cervical cancer cell line exhibited a high similarity of about 96% with circulating extracellular vesicles obtained from the plasma of cervical cancer patients, compared with the expression profile of EVs miRNAs derived from HeLa cell line growing in 2D. On the other hand, they demonstrated that culture and growth conditions do not affect genomic information, carried by EVs secretion, by DNA sequencing analysis (48). Currently, the number of studies focusing in the generation of organoids from primary cervical tissue is scarce. Recently, Lõhmussaar K, and coworkers established a protocol to generate 3D organoids from cervical tissue from both the endocervix and ectocervix that stably recapitulate cervical tissue. These organoids generated differential responses to chemotherapeutic agents, such as carboplatin, cisplatin, and gemcitabine, and grew as xenografts in mice (33, 49).

## 3D Cultures Systems in Ovarian Cancer

Ovarian cancer (OC) is considered the most lethal gynecological cancer due to its high metastatic potential and resistance to chemotherapeutic agents; because of these several studies have been dedicated to demonstrating the importance and influence of 3D cell cultures in the characterization and study of the OC. Due to the above, the need has arisen to develop culture supports and 3D cultures models that allow to mimic the tumor microenvironment to have greater efficiency when testing drugs, this because the use of *in vitro* screening methods on tissue culture polystyrene (TCPS) does not mimic the microenvironment of aggregates *in vivo* when evaluating the response to drugs (50). Additionally, the characteristics genotypic and phenotypic between the different cell lines, can lead to development different morphologies in the formation of spheroids and influence resistance or sensitivity in drug testing, obtaining variable results in the study between 2D and 3D models (51). Hirst and coworkers showed an increase in gene expression associated with hypoxia, drug resistance and stem cell markers in a Multicellular Tumor Spheroids (MCTS) model from epithelial ovarian cancer (EOC), interestingly, they identified that FDA-approved drugs as licofelone and glafenine reversed the gene expression found in MCST (52). The use of different techniques allows to optimize the development of tumor spheroids in OC; the co-culture between OC and mesothelial cells, promotes and facilitates OC spheroid formation in a 3D model, showing a structure of spheroids larger in 3D co-culture than OC cells in a monoculture (53). On the other hand, Angiotensin II (AGII) and its receptor AGTR1 enhanced the formation and increased the growth of OVCA429, and Isogenic highly metastatic OC (HM) tumor spheroids (54). Hydrogel supports made with Poly ethylene glycol-maleimide (PEG-MAL) allow emulation of the omentum, which has made it possible to evaluate resistance to drugs such as Pacitaxel and Mafosfamide as well as the sensitivity to drugs such as Carboplatin, Doxorubicin and LY2606368 in MCTS of SKOV-3 and Ovarian Carcinoma Ascites Spheroids (OCAS) patient-derived models, indicating a greater efficiency and potential use of 3D hydrogel omentum-based MCTS model in drug resistance and sensitivity tests compared with TCPS in OC patients (50). An 3D organotypic model omentum-based using OVCAR4 cells, showed that 3D organotypic models are a suitable tool to evaluate the delivery systems of potential nano-drugs using anti-metastatic nanoparticles with low toxicity such as RAPTA-C [Rutheniumdichloride (p-cymene) PTA] in order to optimize and increase the sensitivity in the treatment of OC (55). On the other hand, Verteporfin, a photosensitizer, was encapsulated efficiently within nanostructured lipid carriers, showing a greater sensitivity to light exposure and a higher cytotoxic effect after treatment in OVCAR3 and SKOV3 spheroids, demonstrating the application of 3D models in the search of photodynamic therapeutic prospects in OC therapy (56). The development and use of organoids models emerged as an alternative in the study of OC due to the limitations in the use of the spheroid models of cell lines. Organoids from EOC, particularly, fallopian tubes and ovarian surface epithelial cells organoids, are the main EOC organoid models used to study ovarian carcinogenesis; however, the lack of microenvironment is one of the principal limitations in the use of organoids because of that several anti-angiogenic, stromal-affecting, and immunotherapy drugs cannot be tested; the combination with the use of microfluid platform, could offer a partial solution to limitation previously mentioned (57).

The tumor microenvironment (TME) is a fundamental part in tumor development and progression, organotypic models consisting of stromal cells, such as human primary mesothelial cells (HPMC) or fibroblasts, and microfluidic models, could be a partial solution to the limitation of 2D culture. Regard 3D organotypic human mesothelium models, the key factors are the use of different ECM proteins and two mainly stromal primary cells, the purpose of this model is to study and identify potential molecules against adhesion, invasion, proliferation, and drug response on OC. The microfluidic 3D model is subjected to a continuous flow of growth factors and nutrients in order to mimic the TME by the flow of peritoneal fluid originated by OC, this model is useful mainly to evaluate the influence of macrophage infiltration in the TME development and its effect on the adhesion, tumorigenicity, proliferation, progression and trancoelomic metastasis in OC (58). The Tumor-associated macrophages (TAM) are key pieces to the survival and proliferation of free detached tumor cells from the primary tumor to form spheroids in early steps of transcoelomic metastasis, Long and coworkers established an *in vitro* spheroid formation assay with a co-culture system composed by GDP+F4/80+CD206+TAMs from an isolated of spheroids of ovarian cancer-bearing donor tomatoIysM-cre mouse, mixed with ID8 cells in a medium with matrigel. The model could support the lack of a tumor microenvironment in 3D models, particularly in the study of the effects of macrophage infiltration on the development and progression tumor (59). Additionally, a hetero-spheroid model, with OVCAR3 OC cells, Ovarian cancer stem cells (CSC) and CD68+ macrophages showed an increase in the expression of CD206, a M2 macrophage marker, IL-10 and WNT5B, in addition to presenting an increase in ALDH+ population and resistance to carboplatin treatment, showing a greater invasive capacity in CDS/M2 spheroids compared with OVCAR3/M0 spheroids, indicating the influence of macrophages in the modulation of the microenvironment of peritoneal fluid in the modulation of the WNT signaling and their relation with the development and progression in OC (60). Ward Rashidi and coworkers showed an increase in the population of ALDH+ from Passage 0 to 6 in an OC 3D hanging drop spheroid model of CSCs; interestingly, an increase in cisplatin resistance was observed in all spheroids serial passage, conversely, a reduction on the cell viability was observed in cells treated with 673A, an ALDH inhibitor, these finding highlights the usefulness of OC 3D model in the study of chemoresistance and tumorigenicity (61).

The gene expression profiles allow the comparative analysis of genotypic and phenotypic features between 2D and 3D cultures, as well as in primary OC tumors. An analysis of transcriptomic profiles in organotypic 3D model identified 1,182 genes differentially expressed. A comparison with primary tumors found 144 common genes that were deregulated in early metastatic colonization. The analysis of cell pathways identified the matrisome, core matrisome, ECM glycoproteins, ECM organization, matrisome associated, focal adhesion and integrin 1 as the main proteins and pathways modulated (62). In the same context, Paullin and coworkers performed a comparative transcriptomic analysis between 2D versus 3D spheroid OC models using HEY cells treated with TGFβ in order to induce the EMT. Results showed a different gene expression profile between models, among them, genes related to chemotherapy resistance (ARK1C1), ECM remodeling (PRSS35) and EMT enhancer transcription factors (SNAI1, SNAI2, ZEB2, TCF3 and SIXI) showed a differential gene expression in 3D compared with 2D culture. In relation to transcriptional networks modulated in 3D spheroids, analysis of the results identified sub-networks that include genes related to response to stress oxidative (PRDX2, CAT, SOD1 and GST01) and transcripts modulated related to heat shock response (HSP90AA1, HSPB1 and HSF1) that may contribute to stabilization of oncogenes and drug resistance (63) (**Figure 3**).

### MicroRNAs Regulation in 3D Ovarian Cancer Cell Cultures

The 3D models can also help to study regulatory mechanisms in OC mediated by miRNAs, in this context, Yoshimura and coworkers evaluated the effect of miR-99a-5p, an microRNA overexpressed in EOC, in peritoneal dissemination, using human peritoneal mesothelial cells (HPMCs) treated with EOC-derived exosomes. Results showed that overexpression of miR-99a-5p in HMPCs promoted the EOC invasion by affecting HPMCs by fibronectin and vitronectin upregulation suggesting that it could be considered as an EOC biomarker in serum and a potential therapeutic target (64). Altogether, reports indicate various advantages of the use of the 3D models culture models for the study and characterization of the development, progression, invasion and treatments of ovarian cancer.

## 3D Cultures Systems in Endometrial Cancer

Endometrial cancer is the most prevalent gynecologic malignancy and a leading cause of morbidity and mortality in females (65). Endometrial cancer has been classified into two main groups, type I or type II, according to their clinicopathologic and molecular characteristics; estrogen-dependent type 1 endometrioid adenocarcinomas account for 80% of cases and are associated with endometrial hyperplasia with characteristic mutations in KRAS, PIK3CA and PTEN whereas type 2 tumors are of non-endometrioid histology, are estrogen-independent, are associated with endometrial atrophy and usually have mutations in TP53 and HER-2 (66, 67). Nowadays, there are several preclinical models in endometrial cancer to evaluate drug efficacy and predict patient outcomes. These include traditional monolayer 2D cultures, organoids, spheroids and animal models. However, some models present limitations (68, 69). For example, Chitcholtan and coworkers demonstrated reduced proliferation in 3D cultures of Ishikawa, RL95-2, EN-1078D and KLE endometrial cancer cell lines compared to 2D cultures, which correlated with decreased expression of the proliferative protein marker PCNA. In addition, altered metabolic phenotypes were observed, including decreased glucose uptake, independent of glucose transporter (GLUT) expression, and down-regulation of vascular epidermal growth factor (VEGF) secretion (70). Together, these data demonstrate that 3D cultures can affect the proliferation and metabolic behavior of endometrial cancer cells compared to 2D cells. Thus, the use of these *in vitro* models to assess the drug response in preclinical trials is important. The 3D cultures of RL95-2 and KLE endometrial cancer cell lines showed increased resistance to doxorubicin compared to 2D cultures, this was due to MAPK inactivation (71). Likewise, it has been shown that deletion of the ETS translocation variant 4 (ETV4), a candidate factor controlling ER genomic binding in endometrial cancer cells, led to decreased growth in 3D cultures of the Ishikawa endometrial cancer cell line (72). Nevertheless, one of the main limitations of these systems is the lack of incorporation of non-epithelial cells, which is why 3D co-cultures are now being developed, incorporating both stromal and epithelial cells. For this reason, 3D co-cultures are now being developed, incorporating both stromal and epithelial cells. These spheroids are phenotypically comparable to endometrial cancer tissue *in vivo*. In other study, Al-Juboori and coworkers performed a proteomic analysis to assess the biological relevance of spheroids in 3D co-culture (HESC/Ishikawa), they found 591 common proteins and canonical pathways that are closely related to endometrial biology in the 3D co-culture model compared to human endometrial tissue (73). On the other hand, the influence of fibroblasts on the invasion of endometrial cancer cells in 3D cultures has been analyzed, showing that Ishikawa endometrial cancer cells co-cultured with fibroblasts in 3D show a high invasion capacity and over-express proteins such as metalloproteinases (MMPs) and plasminogen activators (PA), compared to 3D cultures without fibroblasts (74). Other types of 3D preclinical models relevant to endometrial cancer patients are patient-derived organoids, patient-derived xenografts and patient-derived explants (75). Maru, Y and coworkers established a Matrigel bilayer organoid culture (MBOC) in gynecological tumors, demonstrating that the recovered organoids basically retained the characteristics of the original tumors (76).

## 3D Cultures Systems in Breast Cancer

Breast cancer is a major public health problem due to its high incidence and mortality, being the most common cancer in women worldwide (77). The search for new drugs against breast cancer remains an important field in cancer research. However, the results of the effectiveness of treatments obtained *in vitro* have not been reproduced in the clinic. This is largely because most pre-clinical studies are generated from 2D cultures that do not resemble the “true biology” of the tumor *in vivo* (78, 79). In an early study, dit Faute and coworkers demonstrated that 3D cultures resulted in decreased proliferative rate of both MCF-7 breast cancer cell line and multi-resistant cells (MDR-MCF-7), reduced drug sensitivity of MCF-7 cells, and did not affect the resistance of MDR-MCF-7 cells. In addition, transmission electron microscopy assays demonstrated that MCF-7 cells grown as spheroids had a junctional system involving E-cadherin, tight-junctions and desmosomes, promoting drug resistance. Interestingly, in MCF-7 multi-resistant cell spheroids, cell cohesion was mostly due to membrane interdigitations, induced invasive properties (80). Another study group tested the sensitivity of cisplatin of the MCF-7 cell line grown in 2D and 3D cultures. Similarly, they demonstrated that resistance to cisplatin was mainly generated in 3D cultures which seems to be generated by interactions with the tumor microenvironment. Interestingly, it was demonstrated that 3D-cultured cells were able to progress through the S-cell cycle phase, due to the upregulation of translesion (TLS) DNA polymerase expression and the activation of the ATR-Chk1 pathway. Furthermore, co-treatment with a pharmacological ATR inhibitor (VE-821) generated a response to cisplatin (81).

In other study, Lovitt and coworkers found that spheroids cells displayed more chemoresistance to doxorubicin corresponding to higher IC50 values than conventional monolayer cells in MCF-7 and MDA-MB-23 breast cancer cell lines, mediated by cell-to-ECM interactions. Interestingly, inhibition of integrin signaling in combination with doxorubicin reduced the viability of breast cancer cells (82). Recently, a 3D-μTP culture system was established; it was manufactured by seeding tumor and/or fibroblast cells on biodegradable porous microcarriers in a dynamic culture system (83). Similarly, the efficacy of doxorubicin (DOX) was evaluated in two different 3D cancer models: microtissue (3D-μTP) versus spheroid, both models were formed by co-culturing MCF-7 cell line with fibroblasts. It was demonstrated that the 3D-μTP model showed increased DOX diffusion and decreased cell viability compared to spheroid. Moreover, they demonstrated that, beside multi-cellularity, the presence of a cell-assembled ECM in the 3D-μTP model also played a crucial role in modulating the drug response (84). Another effective treatment for breast cancer is trastuzumab, a recombinant humanized monoclonal antibody targeting human epidermal growth factor receptor 2 (HER2), a gene frequently amplified in 30% of breast cancer cases, and associated with poor prognosis in breast cancer patients (85). Several studies have demonstrated that trastuzumab significantly improved the prognosis of breast cancer patients with HER2 overexpression (86). This is because trastuzumab inhibits several signaling pathways, such as phosphatidylinositol 3-kinase (PI3K)-AKT serine/threonine kinase 1 and mitogen activated protein kinase (MEK)/extracellular signal regulated kinase (ERK) (87). Tatara and coworkers demonstrated that 3D cultures better simulate the cytological and biochemical responses to trastuzumab-induced apoptosis and resistance to trastuzumab associated with the PIK3CA mutation compared to 2D cultures. They observed increased expression of poly (ADP-ribose) polymerase (PARP) cleaved only in PIK3CA-wt lines grown in 3D in response to trastuzumab, but not in PIK3CA-wt or PIK3CA-mt lines grown in 2D (88).

Likewise, Gangadhara and coworkers demonstrated that breast cancer cell lines grown in 3D Matrigel-based culture system showed resistance to trastuzumab compared to 2D cultures, generated by AKT/MAPK extracellular matrix-mediated signaling. Interestingly, MAPK suppression in 3D cultures restoring the therapeutic response (89). Finally, Rodriguez and coworkers demonstrated that the hypoxic environment developed in the spheroids modulates the response to Trastuzumab in the breast cancer cell line HER2+. Furthermore, the acquired resistance to Trastuzumab in 3D cultures was associated with an increase in the population of cancer stem cells (90). Another anti-tumor chemotherapeutic agent is the taxane paclitaxel (Ptx) which binds to- and stabilizes cytoskeleton microtubules resulting in mitosis inhibition (91). Recently, new strategies have been described that allow the drug to accumulate at the site of the tumor and simultaneously decrease the concentration in the rest of the body, thus avoiding serious side effects, such as bone marrow suppression and neurotoxicity (92). This targeted drug delivery can be achieved by magnetic drug targeting (MDT). Lugert and coworkers developed Ptx-functionalized super paramagnetic iron oxide (SPION) nanoparticles coated with lauric acid (LA) and human serum albumin (HSA; SPION LA-HSA-Ptx) and analyzed their efficacy in different breast cancer cell lines cultured in 2D and 3D. They demonstrated that the binding of the antiproliferative and antitumor agent Ptx to the biocompatible and magnetically susceptible carrier SPION LA-HSA was effective in different breast cancer cell lines and did not influence the cytotoxic efficacy of the chemotherapeutic drug. Furthermore, they found not significant differences between the 2D and 3D culture systems (93).

On the other hand, compounds of natural origin with anti-cancer activity have been investigated. An example is Ginger (Zingiber officinale Roscoe). Ginger is the rhizome of plants in the Zingiberaceae family and has been widely used as a medicinal plant for thousands of years, due to its phenolic compounds, [4], [6], [8], and [10]-gingerols (94). It has been demonstrated that gingerols, have multiple anti-cancer effects, inhibiting the cellular proliferation of MDA-MB-231 triple negative breast cancer cells compared to non-tumor cells (95). Therefore, Fuzer and coworkers analyzed the anti-cancer activity of [10]-gingerol in breast cancer HMT-3522 cells growing in lr-ECM in 3D culture. They demonstrated that [10]-gingerol promoted cytotoxicity in linear HMT-3522 (T4-2) cells compared to non-malignant S1 cells. Furthermore, [10]-gingerol induced apoptosis in the HMT-3522 (T4-2) cell line in breast cancer (96).

Interestingly, extracellular matrix signals have been demonstrated to play a crucial role in apoptotic sensitivity in response to chemotherapeutic agents for non-malignant and malignant breast cell lines in 2D and 3D culture (97, 98). This is largely because cells grown in 3D adopt morphologies similar to those of tissues *in vivo*. Kenny and coworkers analyzed the morphological phenotype of 25 of these breast cell lines grown in 2D and 3D cultures, and their gene expression profiles under these same conditions. They demonstrated that breast cancer cell lines grown in 2D did not show different morphologies, however, when grown in 3D they adopted four different morphologies called: Round, Massive, Grape-like and Stellate. Furthermore, the 3D microenvironment produced significant changes in the gene expression profiles of these cancer cell lines (26). In particular, genes encoding proteins involved in signal transduction were over-expressed in 3D cell cultures compared to 2D cultures. Therefore, it is important to understand that cancer is a complex process that depends both on the behavior of the cancer cells and on the function of the non-malignant supporting cells in the tumor microenvironment (99). Tumor-associated mesenchymal stromal cells (TA-MSC) are a major component of the tumor microenvironment; they contribute to cancer progression by promoting metastasis, vascularization of the tumor and contribute to cancer cell resistance to chemotherapy (100). One way to study these TA-MSC is through 3D cell cultures, because cell signaling, and drug responses differ when cells are cultured on rigid 2D substrates or using 3D cell culture systems that more closely mimic the tumor microenvironment (101). Blache and coworkers demonstrated that secretions from the 3D-cultured MDA-MB-231 breast cancer cell line convert mesenchymal stromal cells (MSC) to MSC-AT, generating an immunomodulatory phenotype that is particularly prominent in response to bone-tropic cancer cells (102). The development of 3D cultures allows us to understand the molecular mechanisms of drug resistance and the biology of breast cancer (**Figure 3D**).

### MicroRNAs Regulation in 3D Breast Cancer Cell Cultures

The expression and function of miRNAs in breast cancer cells have long been derived from 2D cultures, which lack the tumor microenvironment. However, recently the expression of miRNAs in 3D versus 2D cultures in different breast cancer cell lines has been described. For example, Nguyen and coworkers analyzed the expression profile of miRNAs in 3D compared to 2D cultures in the MCF-7 (non-invasive) and MDA-MB-231 (invasive) breast cancer cell lines. They showed that 49 miRNAs were differentially expressed in the MCF-7 cell line in 3D cultures compared to 2D, of those 24 were upregulated and 25 were downregulated. Whereas, in the MDA-MB231 cell line, 28 miRNAs were differentially expressed, with 22 miRNAs upregulated and 6 miRNAs downregulated. In addition, two miR-200 family members, miR-141 and miR-429 were overexpressed only in 3D cultures in the MCF-7 cell line. Overexpression of miR-429 in MDA-MB231 cells attenuated their invasive stellate morphology in 3D culture. This suggests that the differential expression profile between the two cell lines is probably due to miRNAs regulating mass morphology in the MCF-7 cell line and invasive stellate morphology in MDA-MB231 cells (25). Furthermore, it has been shown that the expression profiles of miRNAs in MDA-MB-231 cell line cultured in 3D are like the changes reported in highly invasive breast tumors. For example, miR-146a-5p, which regulates cancer progression or miR-210, which is over-expressed in response to hypoxia in breast cancer. Suggesting that 3D cultures better mimic tumors *in vivo* than traditional 2D culture (103).

On the other hand, the use of natural compounds in the treatment of cancer is increasing. Such is the case of silibinin, which is a natural flavonoid, and the anticancer and chemopreventive effects of silibinin have been demonstrated in different types of cancer (104). Yazdi and coworkers analyzed the effect of silibinin on cell viability and miRNA expression in 3D and 2D cultures of the T47D breast cancer cell line. They demonstrated that the 3D cultures show higher drug resistance, similar to what occurs *in vivo* and is largely due to the fact that the cells are in different stages of growth, including proliferation, hypoxia, apoptosis, necrosis and quiescent phase. Furthermore, they demonstrated that silibinin promotes apoptosis in both 3D and 2D cultures. Finally, they demonstrated decreased expression of miR-21, miR-15a, and miR-141, in silibinin-treated cells in 3D and 2D cultures of the T47D cell line (105).

## 3D Cultures Applications in Gynecological and Breast Cancer Translational Research

3D culture models in gynecological and breast cancer provide a valuable platform to investigate the molecular processes leading to uncontrolled cell proliferation and metastasis which may allow for novel drugs discovery. This is of utmost importance, because resistance to chemo- and radiotherapy is common in patients with gynecological and breast cancer, in indeed a large proportion of patients undergo excessively toxic treatments with no or minimal therapeutic benefit (106). The utilization of 3D cultures and organoids will help to predict the responsiveness of patients to treatments and will allow tailoring specific treatments for each patient, resulting in personalized therapies. For example, Boretto, M and coworkers developed organoid cultures derived from endometrial cancer patients as preclinical models for screening drugs, for screening drugs, such as paclitaxel, 5-fluorouracil, carboplatin, doxorubicin) and everolimus (mTOR inhibitor), showed patient-specific responses (107). Furthermore, the STAT3 transcription factor inhibitor, BBI608 (Napabucasin), strongly inhibited the growth of patient-derived organoids through inhibition of growth receptor tyrosine kinase (108).

On the other hand, one of the main characteristics of ovarian cancer is its genetic heterogeneity, so differential responses to drugs in ovarian cancer have to be expected. The development of organoids derived from individual ovarian cancer lines will allow screening for different drugs, for example, the HGS-3.1 organoid line was sensitive to gemcitabine, adavosertib, carboplatin and paclitaxel and resistant to drugs targeting the PI3K/AKT/mTOR pathway, while the HGS-23 line showed a pattern of sensitivity to the opposite drugs. Furthermore, organoids have been demonstrated to capture tumor heterogeneity (106). The radiosensitivity of cervical cancer organoids has been investigated. Nakajima, A and coworkers demonstrated that organoid growth was inhibited in a dose-dependent manner one week after irradiation. Radiosensitivity was patient-specific and matched the response of the xenografted tumor and the patient. Interestingly, hypoxia-inducible factor 1α (HIF-1α) target gene expression was up-regulated in organoids derived from resistant cancer tissues. HIF-1α protein levels increased several hours after irradiation (109). Finally, breast cancer organoids reflect tumor heterogeneity, it has been observed that breast cancer organoids can achieve 60% or more similarity of characteristics and gene profile expression with tumors *in vivo* (110). Furthermore, breast cancer organoids can be used as an effective *in vitro* model for the study of personalized treatment. Garcia-Davis, S and coworkers evaluated the antitumor effect of *laurinterol*, the main compound of an ethanolic extract of *Laurencia johnstonii* on breast cancer organoids. They found a dose-dependent inhibition of metabolic activity, as well as morphological and nuclear changes characteristic of apoptosis. However, they observed a heterogeneous response that was associated with the individual response of each human tumor sample, being associated with intratumoral heterogeneity (111). Recently, Carter and coworkers developed an experimental protocol to infect breast cancer organoid cultures with oncolytic viruses and compared the oncolytic effects of a measles vaccine virus (MeV) and a vaccinia virus (GLV), genetically modified, allowing enzymatic conversion of the 5-fluorocytosine (5-FC) prodrug into the cytotoxic compounds 5-fluorouracil (5-FU) and 5-fluorouridine monophosphate (5-FUMP), to investigate the effects of oncolytic virotherapy. They demonstrated that oncolytic viruses significantly inhibited cell viability in organoid cultures derived from breast cancer tissue. Thus, the model provides a promising *in vitro* method to aid further testing of virotherapeutic vectors for *in vivo* use (112).

At present, breast and gynecological cancer organoids represent an optimal model for the compression of tumor biology, and their applications in the screening of new tumor drugs has significantly contributed to clinical applications (113). However, although promising breast and gynecological cancer organoids have some limitations such as: i) they lack the complete technology to simultaneously connect organoids and tumor microenvironment, ii) the generation of breast and gynecological cancer organoids from patients are mainly surgical tissue or puncture, however, it is believed that some of the cellular heterogeneity of *in vivo* tumors is lost in the sampling process and iii) they lack mesenchymal cells, so they do not have nervous and vascular system, presenting some differences with solid tumors (114, 115). These limitations must be overcome in the immediate future of 3D and organoids technologies to contribute to the advance of the field. In the future, 3D and organoid culture models combined with recent biotechnological progress will offer exciting improvements for the precise application of this technology.

## Conclusions

Although 2D cultures have been used for a long time, they do not reflect the biology of cancer, making them an inefficient model to study the processes associated with cellular responses to chemotherapeutic exposure. On the other hand, the establishment of 3D cultures are potentially a better approach in the search for new biomarkers and new treatment strategies in breast and gynecological cancer, due to their physiological relevance bringing us closer to the goal of personalized medicine. The main contributions of 3D cultures in gynecological and breast cancer can be summarized as follows i) they have improved our understanding of cancer biology, ii) they have helped to better understand the molecular mechanisms of drug resistance by the identification on novel players in these processes, iii) they capture phenotypic heterogeneity, iv) they modify gene expression and cell behavior in a similar way to *in vivo* condition, v) and they mimic the tumor micro-environment in a similar way to *in vivo* tumors. In conclusion the development of 3D culture models in breast and gynecologic cancer will help to further understand cancer biology and to develop new drugs and predict drug response to address poor response rates and improve survival outcomes in patients.

## Author Contributions

CL-C, YS-V, JV, LM, and RR-P conceived and designed the review. CL-C, YS-V, JV, YP-N, GM-L, LM, RR-P, and SN-O performed the literature review and were involved in the writing and revision of the manuscript. YS-V, YP-N, CP-P, and GM-L prepared all the figures. All authors have read and agreed to the published version of the manuscript.

## Funding

This research was funded by Consejo Nacional de Ciencia y Tecnologia (CONACYT), Mexico, Grant FORDECYT-PRONACES/51207/2020. Convenio I1200/189/2020. YS-V and SN-O received scholarships 754782 and 789774, respectively, from CONACYT, Mexico.

## Conflict of Interest

The authors declare that the research was conducted in the absence of any commercial or financial relationships that could be construed as a potential conflict of interest.

## Publisher’s Note

All claims expressed in this article are solely those of the authors and do not necessarily represent those of their affiliated organizations, or those of the publisher, the editors and the reviewers. Any product that may be evaluated in this article, or claim that may be made by its manufacturer, is not guaranteed or endorsed by the publisher.
